# Cross-suppression of EGFR ligands *amphiregulin* and *epiregulin* and de-repression of FGFR3 signalling contribute to cetuximab resistance in wild-type *KRAS* tumour cells

**DOI:** 10.1038/bjc.2012.103

**Published:** 2012-04-10

**Authors:** C Oliveras-Ferraros, S Cufí, B Queralt, A Vazquez-Martin, B Martin-Castillo, R de Llorens, J Bosch-Barrera, J Brunet, J A Menendez

**Affiliations:** 1 Unit of Translational Research, Catalan Institute of Oncology (ICO), Girona, Spain; 2 Girona Biomedical Research Institute (IDIBGi), Girona, Spain; 3 Department of Medical Oncology, Catalan Institute of Oncology (ICO), Girona, Spain; 4 Unit of Clinical Research, Catalan Institute of Oncology (ICO), Girona, Spain; 5 Department of Biology, Biochemistry and Molecular Biology Unit, University of Girona, Girona, Spain

**Keywords:** cetuximab, KRAS, EGFR, amphiregulin, epiregulin, FGFR3

## Abstract

**BACKGROUND:**

In addition to the mutational status of *KRAS*, the epidermal growth factor receptor (EGFR) ligands amphiregulin (*AREG*) and epiregulin (*EREG*) might function as *bona fide* biomarkers of cetuximab (Ctx) sensitivity for most EGFR-driven carcinomas.

**METHODS:**

Lentivirus-delivered small hairpin RNAs were employed to specifically reduce *AREG* or *EREG* gene expression in wild-type *KRAS* A431 squamous cell carcinoma cells. Colony-forming assays were used to monitor the impact of *AREG* and *EREG* knockdown on Ctx efficacy. Amphiregulin and EREG protein expression levels were assessed by quantitative ELISA in parental A431 cells and in pooled populations of A431 cells adapted to grow in the presence of Ctx. A phosphoproteomic platform was used to measure the relative level of phosphorylation of 42 distinct receptor tyrosine kinases before and after the acquisition of resistance to Ctx.

**RESULTS:**

Stable gene silencing of either ligand was found to notably reduce the expression of the other ligand. Parental A431 cells with normal expression levels of *AREG/EREG* exhibited significantly increased growth inhibition in response to Ctx, compared with derivatives that are engineered to produce minimal *AREG/EREG*. The parental A431 cells acutely treated with Ctx exhibited reduced basal expression levels of AREG/EREG. Pooled populations of Ctx-resistant A431 cells expressed significantly lower levels of AREG/EREG and were insensitive to the downregulatory effects of Ctx. Phosphoproteomic screen identified a remarkable hyperactivation of FGFR3 in Ctx-resistant A431 cells, which gained sensitivity to the cytotoxic and apoptotic effects of the FGFR3 TK inhibitor PD173074. The A431 parental cells acutely treated with Ctx rapidly activated FGFR3 and their concomitant exposure to Ctx and PD173074 resulted in synergistic apoptosis.

**CONCLUSION:**

Cross-suppression of *AREG/EREG* expression may explain the tight co-expression of *AREG* and *EREG*, as well as their tendency to be more highly expressed than other EGFR ligands to determine Ctx efficacy. The positive selection for Ctx-resistant tumour cells exhibiting AREG/EREG cross-suppression may have an important role in the emergence of Ctx resistance. As de-repression of FGFR3 activity rapidly replaces the loss of EGFR-ligand signalling in terms of cell proliferation and survival, combinations of Ctx and FGFR3-targeted drugs may be a valuable strategy to enhance the efficacy of single Ctx while preventing or delaying acquired resistance to Ctx.

The messenger RNA (mRNA) levels of the epidermal growth factor receptor (EGFR) ligands *amphiregulin* (*AREG*) and *epiregulin* (*EREG*) may be potential predictors of tumour response to cetuximab (Ctx, Erbitux, Branchburg, NJ, USA) in metastatic colorectal cancer (mCRC) patients harbouring wild-type (wt) *KRAS* tumours. A pioneering pharmacogenomic approach in pre-treatment biopsy samples from metastatic sites showed that high expression levels of *AREG* and *EREG* mRNA were highly predictive of the clinical outcome after Ctx monotherapy in mCRC (Khambata-Ford *et al*, 2007). Jacobs *et al* (2009) confirmed the strong association between increased *AREG* and *EREG* gene expression, and increased tumour response and patient survival after Ctx treatment in mCRC; high levels of *AREG* and *EREG* mRNAs in the primary tumour were positively associated with increased responsiveness to Ctx treatment in metastatic disease. Assessment of the predictive effect of (1) high *vs* low *EREG* expression among wt *KRAS* patients and (2) high *EREG* and a wt *KRAS* status (‘combimarker’) *vs* all other patients on the overall survival and progression-free survival indicated that mCRC patients with wt *KRAS* and high *EREG* gene expression exhibited significantly larger Ctx treatment effects (Jonker *et al*, 2009). The mRNA expression levels of the MAPK phosphatases *DUSPs* (*4* and *6*), which encode phosphatase inhibitors of the EGFR pathway, may be negative indicators of the outcome after Ctx treatment in mCRC (De Roock *et al*, 2009). *DUSPs* were originally identified as resistance markers to Ctx in unselected patients, and the use of a four-gene expression model including *AREG*, *EREG,* and *DUSP6* (as well as Solute Carrier Family 26 member 3, *SLC26A3*) was suggested to improve the identification of Ctx responders among pre-selected wt *KRAS* mCRC patients (Khambata-Ford *et al*, 2007; Baker *et al*, 2008).

The autocrine production of *AREG* has been described as an important biomarker associated with enhanced growth inhibition by Ctx in non-small-cell lung cancer (NSCLC) cell lines and in NSCLC patients (Yonesaka *et al*, 2008). Our recently described transcriptome signature for Ctx efficacy in cultured models of squamous cell carcinomas (SCCs) revealed that the molecular function of Ctx depended on high expression levels of genes encoding EGFR ligands (such as *AREG* and *EREG*) concomitant with downregulation of *DUSP*-regulated EGFR inhibition (Oliveras-Ferraros *et al*, 2011a, 2011b). A dramatic downregulation of *AREG/EREG* mRNA expression, but not of other EGFR ligands, has been found to correlate with loss of Ctx efficacy (Oliveras-Ferraros *et al*, 2011a, 2011b). Therefore, we sought to establish the following: (a) whether cross-regulation mechanisms can explain the impact of *AREG/EREG* on Ctx efficacy; (b) whether the loss of *AREG* or *EREG* is sufficient to fully establish tumour resistance to Ctx; (c) whether the downregulation of AREG/EREG expression is indispensable for the Ctx mechanism of action; and/or (d) whether kinase-switching phenomena might contribute to bypass loss of EGFR-ligand signalling caused by Ctx. Here, we present the first evidence that AREG/EREG cross-suppression (i.e., the downregulation of a gene through the inhibition of a related gene) is a previously unrecognised phenomenon that can explain the tight co-expression of AREG and EREG and the tendency of AREG and EREG to be more highly expressed than other EGFR ligands to determine the efficacy of Ctx. Additionally, we provide the first evidence that aberrant overactivation of FGFR3 rapidly and efficiently bypasses EGFR signalling upon loss of AREG/EREG. Our findings confirm that minimal expression of *AREG/EREG* might identify wt *KRAS* tumours that have a high likelihood of resistance to Ctx and strongly suggest that positive selection for Ctx-resistant tumour cells exhibiting *de novo* or induced AREG/EREG cross-suppression most likely has an important role in determining the emergence of Ctx resistance. The finding of EGFR/FGFR3 kinase switching and acquired FGFR3 pro-survival signalling suggest investigation of new combinations of Ctx with selective inhibitors of FGFR3 to prevent or delay acquired resistance to Ctx.

## Materials and methods

### Culture conditions

Parental A431 vulvar squamous carcinoma cells (obtained from the American Type Culture Collection, Manassas, VA, USA) were routinely grown in Dulbecco’s modified Eagle’s medium (DMEM, Gibco Cell Culture Systems, Invitrogen S.A., Barcelona, Spain) containing 10% heat-inactivated foetal bovine serum (FBS, Bio-Whittaker, Inc., Walkersville, MD, USA), 1% ℒ-glutamine, 1% sodium pyruvate, 50 U ml^−1^ penicillin, and 50 U ml^−1^ streptomycin. The cells were maintained at 37 °C in a humidified atmosphere with 5% CO_2_. The cells were periodically screened for *Mycoplasma* contamination.

### Drugs

Cetuximab was kindly provided by the Hospital Universitari de Girona Dr Josep Trueta Pharmacy (Girona, Spain). Cetuximab was solubilised using 10 mmol l^−1^ NaCl in phosphate buffered saline (PBS) at pH 7.2 in bacteriostatic water for injection purposes (stock solution at 2 mg ml^−1^), stored at 4 °C and used within 1 month of preparation. PD173074 was purchased from Sigma (St Louis, MO, USA). A 10 mmol l^−1^ concentrated stock solution of PD173074 was prepared by reconstituting the entire contents of the vial in an adequate volume of DMSO.

### Immunoblotting procedures

Western blots were performed using an SDS–PAGE electrophoresis system as described previously (Oliveras-Ferraros *et al*, 2011a, 2011b), employing anti-AREG (Santa Cruz Biotechnology, Santa Cruz, CA, USA, cat. no. sc-5796), anti-EREG (Abcam, Cambridge, MA, USA, cat. no. ab89291), or anti-DUSP6 (Abcam, cat. no. ab54940) antibodies, as specified. Immunoblotting experiments were repeated at least three times, and the blots were re-probed for *β*-actin (Santa Cruz Biotechnology, cat. no. sc-47778) to control for protein loading and transfer. Densitometric values of protein bands were quantified using Scion Image software (Scion Corporation, Frederick, MD, USA).

### Establishment of long-term (LT) Ctx-adapted *wt KRAS* tumour cell populations

Beginning with the IC_50_ of Ctx (25 *μ*g ml^−1^) against parental A431 cells, the exposure dose of Ctx was progressively increased every 2–3 weeks until a four-dose doubling had been successfully achieved. Parental control cells were cultured strictly in parallel and exposed to the PBS vehicle. This approach resulted in the establishment of two LT Ctx-adapted A431 POOLs, which were then maintained in continuous culture with the maximal achieved dose of Ctx. When challenged with Ctx at doses as high as 200 *μ*g ml^−1^, the LT-Ctx A431 POOL1/2 retained a >90% active metabolic status as assessed by MTT-based cell viability assays compared with Ctx-naive A431 parental cells, which exhibited a >50% reduction in viability.

### Stable silencing of EGFR ligands and DUSP6

Stable A431-derived cell lines expressing small hairpin RNAs (shRNAs) against *AREG*, *EREG* or *DUSP6* (*MKP3*) were generated by lentiviral infection according to the manufacturer’s instructions (Santa Cruz Biotechnology). Lentiviruses expressing a scrambled shRNA were used as a negative control (mock).

### Quantitative determination of AREG and EREG levels

The human AREG ELISA kit from Ray Biotech, Inc. (Norcross, GA, USA, cat. no. ELH-AR001) and the human *EREG* ELISA kit from Uscn Life Science Inc. (Wuhan, China, cat. no. E91945Hu) were used for the quantitative determination of AREG and EREG expression levels, respectively, in whole-cell lysates, following the manufacturer’s instructions.

### Colony formation

Stable A431-derived cell lines expressing shRNAs against *AREG* and *EREG* or expressing a scrambled shRNA (control) were cultured in six-well plates at a density of 1000 cells per well (in triplicate) and incubated for 18 h to allow for attachment. The cells were then treated with regular medium in the absence or presence of 100 *μ*g ml^−1^ of Ctx for 3 days; the cells were allowed to grow in a drug-free medium for an additional 10 days before the colonies were stained with crystal violet.

### Phosphoproteome profiling

The cells were rinsed with cold-PBS and immediately solubilised in NP-40 lysis buffer (1% NP-40, 20 mmol l^−1^ Tris-HCl (pH 8.0), 137 mmol l^−1^ NaCl, 10% glycerol, 2 mmol l^−1^ EDTA, 1 mmol l^−1^ sodium orthovanadate, 10 *μ*g ml^−1^ aprotinin, 10 *μ*g ml^−1^ leupeptin) by rocking the lysates gently at 4 °C for 30 min. Following microcentrifugation at 14 000 × **g** for 5 min, supernatants were transferred into a clean test tube and sample protein concentrations were determined using the BCA Protein kit (Pierce, Rockford, IL, USA). Lysates were diluted and incubated with Human Phospho-RTK and Human Phospho-MAPK Arrays (Proteome Profiler; R&D Systems; Minneapolis, MN, USA) as per the manufacturer’s instructions. In this method, capture and control antibodies have been spotted in duplicate on nitrocellulose membranes. Briefly, the membranes were blocked with 5% bovine serum album (BSA)/TBS (0.01 mol/l Tris-HCl, pH 7.6) for 1 h. Membranes were then incubated with 750 *μ*g of total protein. After extensive washing with TBS including 0.1% v/v Tween-20, three times for 5 min, to remove unbound materials, the membranes were then incubated with HRP-phospho-receptor tyrosine kinase (RTK) or phospho-MAPK antibodies for 2 h at room temperature (RT). Unbound HRP antibody was washed out with TBS including 0.1% v/v Tween-20. Finally, array data were developed on an X-ray film using a chemiluminescence detection system (Amersham Life Sciences, Piscataway, NJ, USA).

### Metabolic status assessment (MTT-based cell viability assays)

The ability of Ctx, PD173074, and Ctx+PD173074 to affect cell viability was determined using a standard colorimetric MTT (3-4,5-dimethylthiazol-2-yl-2,5-diphenyl-tetrazolium bromide) reduction assay. The MTT assay was done as described previously (Oliveras-Ferraros *et al*, 2011a).

### Apoptosis assays

The ability of Ctx, PD173074, and Ctx+PD173074 to induce apoptosis was assessed using the Cell Death Detection ELISA^PLUS^ Kit obtained from Roche Diagnostics (Barcelona, Spain). Briefly, cells (5–10 × 10^3^ cells per well) were grown in 96-well plates and treated, in triplicates, for 72 h with the indicated doses of Ctx, PD173073 or Ctx+PD173073, as specified. The supernatant was discharged, lysis buffer was added, and the samples were incubated at RT for 30 min following the manufacturer's instructions. Anti-histone biotin and anti-DNA peroxidase antibodies were added to each well and incubated at RT for 2 h. After three washes, the peroxidase substrate was added to each well, and the plates were read at 405 nm at multiple time intervals. The enrichment of histone–DNA fragments in treated cells was expressed as fold increase in absorbance as compared with control (vehicle-treated) cells using the following formula: [*A*_405_−*A*_490_]_TREATED_/[*A*_405_−*A*_490_]_UNTREATED_.

### Statistics

The means from ⩾3 groups were compared by ANOVA. The existence of individual differences, in cases of significant *F* values with ANOVA, was tested by Scheffé’s multiple comparisons. In all cases, statistical analyses were performed with XLSTAT (Addinsoft, New York, NY, USA), and *P*<0.05 was considered to be significant.

## Results

### RNAi stably reduces AREG, EREG and DUSP6 expression in A431 SCC cells

**Figure 1:**
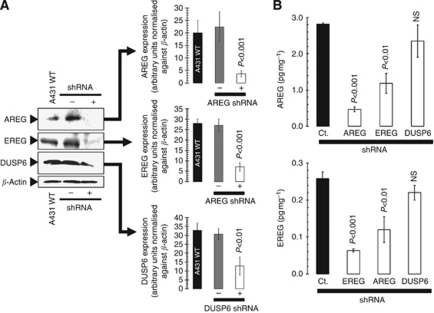
Cross-regulation of EGFR ligands AREG and EREG in A431 SCC cells. (**A**) Lysates from A431 parental cells and their derivatives ‘control shRNA’, ‘AREG shRNA’, ‘EREG shRNA’, and ‘DUSP6-shRNA’ were prepared and analysed by western blot for expression of AREG, EREG, and DUSP6 proteins using specific antibodies as described in the ‘Materials and Methods’ section. The Western blots were also probed with an anti-*β*-actin antibody to demonstrate equal protein loading. (**B**) Commercially available ELISAs were used for the quantitative determination of AREG and EREG protein expression in whole-cell lysates from A431-derived ‘control (Ct.) shRNA’, ‘AREG shRNA’, ‘EREG shRNA’, and ‘DUSP6-shRNA’ cell lines. Results are means (*columns*) and 95% confidence intervals (*bars*) of two independent experiments made in duplicate. Statistically significant differences (one-factor ANOVA analysis) between experimental condition groups (i.e., A431 cells stably expressing shRNAs against AREG, EREG, and DUSP6) and ‘control (Ct.) shRNA’ cells are shown (NS no statistically significant).

Western blot analysis was used to confirm the reduced protein expression of AREG, EREG and DUSP6 in A431 tumour cells stably transduced with *AREG*-, *EREG-*, and *DUSP6*-short hairpin (sh)RNAs. Lysates were isolated from A431 cell cultures that were stably expressing shRNA against AREG, EREG, or DUSP6 and from A431 cells stably transduced with an empty vector (control shRNA). AREG, EREG, and DUSP6 protein expression from these lysates was compared with the expression in the non-transduced parental A431 cells as a control. Immunoblot analyses indicated that AREG and EREG protein levels were reduced to almost undetectable levels (>75% reduction) in the cells stably transduced with the AREG and EREG shRNA plasmids, respectively, when compared with either the control or control shRNA cells (Figure 1A). A431 cells stably transduced with a DUSP6-shRNA plasmid exhibited a less dramatic but still highly significant reduction in DUSP6 protein levels (∼60% reduction). We continued further experiments using stable cell lines each designated as ‘control shRNA’, ‘AREG shRNA’, ‘EREG shRNA’, and ‘DUSP6-shRNA’.

### The EGFR ligands AREG and EREG cross-regulate

To determine whether *AREG* and *EREG* genes are reciprocally regulated, we performed ELISA-based assays to quantitatively monitor the effect of shRNA-mediated knockdown of *AREG* and *EREG* on the expression status of *EREG* and *AREG*, respectively (Figure 1B). Stable gene silencing using lentiviral-delivered shRNAs revealed that when AREG protein expression was specifically downregulated via transduction with *AREG* shRNA, EREG protein expression also decreased by >50%. Likewise, when EREG protein expression was specifically downregulated via transduction with *EREG* shRNA, AREG protein expression concomitantly decreased by ∼60%. By contrast, the expression levels of AREG and EREG remained largely unaltered when DUSP6 protein expression was downregulated with *DUSP6*-shRNA.

### Loss of AREG/EREG negatively impacts Ctx efficacy

**Figure 2:**
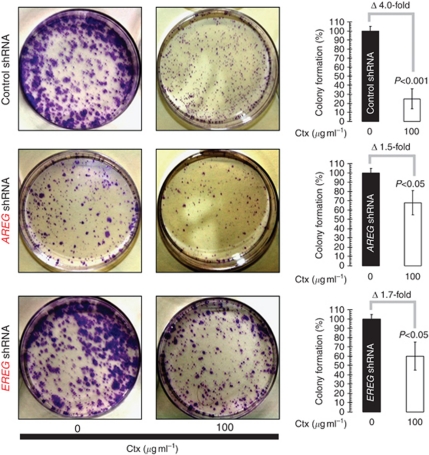
Impact of. *AREG*, *EREG*, or *DUSP6* gene silencing on Ctx-regulated SCC cell proliferation and survival in anchorage-dependent conditions. The left side figure shows representative microphotographs of colonies formed on the plates of control (untreated) and Ctx-treated ‘control (Ct.) shRNA’, ‘AREG shRNA’, or ‘EREG shRNA’ cell cultures, as specified. The right side bar graphs show the number of colonies formed after treatment with Ctx compared with the control (untreated) ‘control (Ct.) shRNA’, ‘AREG shRNA’, or ‘EREG shRNA’ cell cultures, as specified. The error bar indices the 95% confidence intervals for each mean (*columns*) generated from triplicate experiments. Statistically significant differences (one-factor ANOVA analysis) between experimental condition groups (i.e., Ctx-treated) and control (i.e., Ctx-untreated) groups are shown.

To determine whether the loss of the EGFR ligands was sufficient to significantly impact Ctx efficacy, we examined the colony-forming potential of A431 cells before and after the stable knockdown of *AREG* and *EREG* (Figure 2). The growth inhibition by Ctx of A431 control shRNA cells that expressed baseline AREG/EREG protein levels was significantly higher compared with the growth inhibition of cells expressing minimal levels of AREG or EREG. Cetuximab treatment drastically diminished (by approximately 80%, i.e., approximately fourfold) the number of colonies formed by A431 (parental) cells, whereas stable knockdown of the *AREG* or *EREG* genes significantly prevented the Ctx-induced decrease in cell proliferation and survival to less than twofold (Figure 2). It should be noted that when *AREG* expression was targeted, colony formation significantly decreased compared with when *AREG* expression was not targeted in the shRNA-scrambled-transduced A431 cells.

### Downregulation of AREG/EREG is indispensable for the molecular function of Ctx

**Figure 3:**
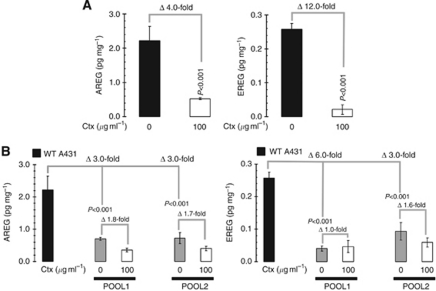
Differential regulation of EGFR ligands AREG and EREG by Ctx in Ctx-responsive and Ctx-resistant SCC cells. AREG- and EREG-specific ELISAs were used to quantitatively assess the effects of Ctx treatment (48 h) in Ctx-responsive A431 parental cells (**A**) and in two independent Ctx-resistant A431 pooled populations (**B**). Results are means (*columns*) and 95% confidence intervals (*bars*) of two independent experiments made in duplicate. Statistically significant differences (one-factor ANOVA analysis) between Ctx-untreated and Ctx-treated cells and/or between A431 parental cells and their Ctx-unresponsive pooled populations are shown.

Based on the assessment results summarised above, it is reasonable to suggest that EGFR ligands AREG/EREG are indicators of a Ctx-responsive EGFR pathway. If this molecular scenario is accurate, then the following predictions can be made: (1) treatment with Ctx should downregulate AREG/EREG expression to elicit Ctx-induced cell growth inhibition, and (2) resistance to Ctx should be accompanied by a loss of Ctx-induced downregulation of AREG/EREG. Culturing of A431 parental tumour cells with Ctx for 48 h remarkably diminished the expression of AREG/EREG proteins to very low or almost undetectable levels (Figure 3A). The ability of Ctx to reduce the protein expression of both AREG (by fourfold) and EREG (by 12-fold) in Ctx-responsive A431 cells was lost upon acquisition of Ctx resistance. First, acquired Ctx resistance was accompanied by significant reductions in the steady-state protein levels of AREG (up to a threefold decrease) and EREG (up to a sixfold decrease). Second, Ctx challenge in pooled populations of Ctx-resistant A431 cells failed to induce significant downregulatory effects in the already compromised AREG/EREG expression levels (Figure 3B).

### Activation of FGFR3 replaces Ctx-induced loss of EGFR-ligand signalling

**Figure 4:**
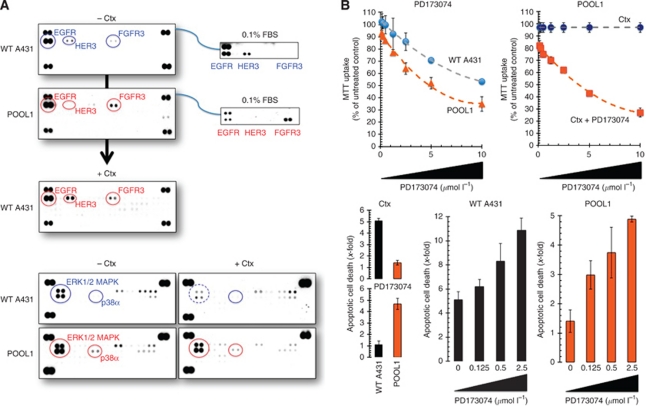
Activation of FGFR3 replaces loss of EGFR-ligands signalling and contributes to Ctx resistance through pathway redundancy. (**A**) Phosphoproteome profiling of A431 cells during acquisition of resistance to Ctx. Total cell lysates (750 *μ*g) from A431 cells before (WT) and after (POOL1) adaptation to Ctx treatment were incubated with membranes of the phosphoproteomic platforms human Phospho-RTK (top panels; 42 different RTKs) and human Phospho-MAPK (bottom panels; 23 different MAPKs and other serine/threonine kinases) as per the manufacturer’s instructions (Proteome Profiler; R&D Systems). Figure shows representative phosphoproteome analyses that were developed on X-ray film following exposure to chemiluminescent reagents. Equivalent results were obtained in three independent experiments. (**B**) Synergy analyses of the interaction between Ctx and PD173074. *Top*: Ctx-naive A431 parental cells and Ctx-resistant POOL1 cells were incubated with graded concentrations of PD173074 in the absence or presence of 100 *μ*g ml^−1^ Ctx, as specified. Cell viability, measured using MTT uptake assays, was expressed as % of untreated control cells (=100% cell viability). Results are means and 95% confidence intervals (*bars*) of three independent experiments made in triplicate. *Bottom*: Quantification of apoptosis-related cell death in Ctx-naive A431 parental cells and Ctx-resistant POOL1 cells in response to 72 h treatment with Ctx, PD173074 or Ctx *plus* PD173074, as specified, was determined by Cell Death ELISA as described in ‘Materials and Methods’. Data are the mean (columns) and 95% confidence intervals (bars) of three independent experiments performed in duplicates.

We decided to investigate whether aberrant activation of EGFR-related HER family members (i.e., HER2, HER3, and HER4) and/or other RTKs might be bypassing chronic loss of EGFR-ligand signalling induced by Ctx. To concurrently assess the activation status of multiple RTKs, we employed a phosphoproteome platform that allows the rapid, sensitive, and simultaneous detection of multiple RTKs in a single antibody-based microarray. Phospho-RTK profiling of cell lysates from untreated A431 parental cells clearly confirmed that this SCC model largely depends on EGFR activation to proliferate as EGFR was the sole receptor exhibiting enhanced phosphorylation among 42 different RTKs (Figure 4A, top). A much weaker phosphorylation of HER3 and FGFR3 could be also detected in A431 parental cells. Of note, Ctx-resistant A431 cells notably retained high levels of EGFR activity while acquiring a remarkable hyperphosphorylation of FGFR3 (Figure 4A, top). To evaluate whether gaining of FGR3 activity was affected by the presence or absence of fetal bovine serum, as this is a rich source of growth factors, a phosphoproteomic screen was carried out in cells growing under low (0.1%)-serum conditions. In the absence of serum, A431 parental cells showed a significant increase in HER3 phosphorylation while the weak, basal phosphorylation of FGFR3 decreased to an undetectable level (Figure 4A, top). In marked contrast, Ctx-resistant A431 cells become even more addicted to FGFR3 signalling as they demonstrated decreased levels of EGFR activity accompanied by a striking hyperactivation of FGFR3 (Figure 4A). In this scenario, it was relevant to evaluate the phosphorylation status of downstream intracellular kinases in response to Ctx before and after the acquisition of refractoriness to Ctx. The A431 parental cells exhibited significant activation of ERK1/ERK2, which was drastically reduced in response to Ctx treatment (Figure 4A, bottom). Conversely, constitutive hyperactivation of ERK1/ERK2 remained largely unaltered to Ctx treatment in Ctx-resistant A431 cells (Figure 4A, bottom). Therefore, loss of EGFR-ligand signalling in Ctx-resistant A431 cells is replaced by aberrant FGFR3 activity and signalling through this receptor can activate the MEK–ERK pathway in a Ctx-independent manner.

### The FGFR3 TK inhibitor PD173074 synergistically enhances the pro-apoptotic effects of Ctx

When A431 parental cells and a pooled population of Ctx-resistant A431 cells were treated with increasing concentrations of PD173074, a small-molecule FGFR3 selective tyrosine kinase inhibitor (TKI), MTT-based cell viability assays failed to detect a half-maximal effective concentration (IC_50_) value in Ctx-responsive A431 parental cells (Figure 4B, top). Conversely, IC_50_ values of less than 5 *μ*mol l^−1^ were obtained in Ctx-resistant A431 cells cultured in the presence of PD173074. Although combined treatment with Ctx and PD173074 notably reduced cell proliferation in pooled populations of Ctx-resistant A431 cells, the lack of any dependency on EGFR-ligand signalling mostly precluded the occurrence of highly significant supra-additive (i.e., synergistic) interactions between the anti-EGFR antibody Ctx and the FGFR3 TKI PD173074 (Figure 4B, top). To determine if these findings related to changes in apoptotic cell death we measured apoptosis-induced DNA–histone fragmentation by a Cell Death ELISA kit, with the values of untreated control cells as onefold. Cetuximab, as single agent, failed to induce significant levels of apoptotic cell death in Ctx-resistant A431 cells; conversely, significantly increased levels of apoptotic cell death were observed in Ctx-resistant A431 cells treated with PD173074 as single agent (up to a fivefold increase in the presence of 10 *μ*mol l^−1^ PD173074). The combined treatment with Ctx and sub-optimal concentrations of PD173074 showed weakly synergistic to nearly additive interactions for augmenting apoptotic cell death in Ctx-resistant A431 cells.

A431 parental cells were likewise sensitive to the pro-apoptotic effects of Ctx (up to a fivefold increase in the presence of 100 *μ*g ml^−1^ Ctx) whereas they remained mostly insensitive to any pro-apoptotic activity of PD173074 (Figure 4B, bottom). Interestingly, A431 parental cells exhibited the highest extent of apoptotic cell death following concurrent exposure to Ctx and PD173074 (up to an 11-fold increase when combining 100 *μ*g ml^−1^ Ctx and 2.5 *μ*mol l^−1^ PD173074; Figure 4B, bottom). As these results indicated that combined treatment with Ctx *plus* PD173072 was more effective than single-modality treatment, we hypothesised that in A431 parental cells in which EGFR is dominant for Ctx-responsive growth signalling, Ctx-induced acute loss of EGFR-ligand signalling might rapidly promote activation of FGFR3 signalling. Parental, Ctx-naive A431 cells were treated with Ctx for 48 h and then lysed and immediately analysed for changes in RTK phosphorylated signalling, which likely represent immediate responses by SCC tumour cells to Ctx. Phospho-RTK profiling likewise confirmed that induction of FGFR3 activity occurs quickly in response to Ctx (Figure 4A, bottom).

## Discussion

With the goal of creating a clinically useful test to dichotomise wt *KRAS* mCRC patients with respect to Ctx sensitivity, Baker *et al* (2011) recently examined a set of 110 biologically based candidate gene expression biomarkers in primary tumour specimens from wt *KRAS* mCRC patients who had been treated with Ctx monotherapy. Their final model that yielded a predictive score included the *AREG*, *EREG*, *DUSP6*, and *SLC26A3* genes, which together sharply dichotomised patients with respect to the likelihood of response to Ctx. This four-gene classification, when used in conjunction with the *KRAS* mutational status, was significantly associated with several indices of clinical outcome compared with use of either expression alone or *KRAS* mutation status alone (Baker *et al*, 2011). Although Baker’s finding that increased *AREG*/*EREG* expression was positively associated with an enhanced sensitivity to Ctx, and other studies likewise agree with the notion that stimulators of the EGFR pathway (e.g., EGFR ligands) are expected to associate with an increased likelihood of disease control in patients receiving Ctx (Khambata-Ford *et al*, 2007; Jacobs *et al*, 2009), none of the above-mentioned studies have established a *causal* relationship between the expression status of *AREG/EREG* and Ctx responsiveness in wt *KRAS* tumour cells. Baker’s study confirmed that unlike the *AREG*/*EREG* genes, the genes that encode other EGFR ligands such as *TGFα*, *HB-EGF*, and *EGF* were not consistently associated with patient outcome, and *AREG*/*EREG* failed to co-express with other significant genes. These findings strongly suggested that *AREG* and *EREG* are tightly co-expressed and that they tend to be more highly expressed than other EGFR ligands in mCRC. However, we have lacked experimental evidence to establish the cross-suppression of *AREG/EREG* as a crucial phenomenon underlying Ctx efficacy. Moreover, it could be argued that multi-gene classifiers incorporating the expression status of *AREG*/*EREG* will be informative of Ctx responsiveness in wt *KRAS* mCRC but not in EGFR-dependent carcinomas with either intact *KRAS* signalling (e.g., head and neck SCC (HNSCC)) or cases in which *KRAS* mutations do not predict a clinical benefit from Ctx (e.g., (NSCLC)) (Mehra *et al*, 2008; Ettinger, 2010; Vincenzi *et al*, 2010). We are beginning to accumulate evidence that *AREG*/*EREG* behave as universal markers for predicting the efficacy of Ctx across most types of EGFR-driven human tumours. In this regard, Yonesaka *et al* (2008) reported that AREG significantly stimulates the growth of Ctx-sensitive NSCLC cells, the sole inhibition of AREG (either by a neutralising antibody or by siRNA) is sufficient to inhibit NSCLC cell growth, and AREG expression (mRNA or protein) is an appropriate biomarker for identifying patients who will benefit from Ctx treatment in both NSCLC and HNSCC.

Studies from our group have used the highly aggressive EGFR-dependent human epidermoid carcinoma A431 cells (four million EGFR receptors per cell; Hsieh *et al*, 2010) as an *in vitro* platform to assess the role of EGFR ligands on the functioning and efficacy of Ctx in SCC. These studies have recently confirmed that the occurrence of a positive feedback loop on EGFR activation driven by genes encoding EGFR ligands and the lack of negative feedback on MAPK activation regulated by DUSPs are both necessary to elicit anti-proliferative responses to Ctx (Oliveras-Ferraros *et al*, 2011a, 2011b). In addition, gene expression signatures for EGFR ligands distinctively relates to the unresponsiveness to mono-HER1 inhibitors (i.e., Ctx), mono-HER2 inhibitors (i.e., trastuzumab) and dual-HER1/HER2 inhibitors (i.e., lapatinib), with minimal overlap between them (Oliveras-Ferraros *et al*, 2011a, 2011b). Therefore, the molecular function of Ctx appears to largely depend on the specific overproduction of the mRNAs encoding AREG and EREG but not those encoding EGF, TGF*α*, or HB-EGF. We now confirm at the protein level that the molecular function of Ctx against SCC cells necessarily depends on the overproduction of the EGFR ligands AREG and EREG. Using shRNA-based approaches, we provide evidence to suggest that AREG/EREG cross-suppression is a pivotal phenomenon that can explain why AREG and EREG tightly co-express and tend to be more highly expressed than other EGFR ligands to determine Ctx efficacy in a clinical setting. We acknowledge that specific knockdown of *AREG* or *EREG* expression failed to completely prevent Ctx-induced inhibition of tumour cell growth and survival. One explanation is that it is difficult to fully neutralise the binding of an autocrine growth factor to its target receptor, whereas an inhibitor, such as a monoclonal antibody, that directly targets the receptor is far more efficient. Therefore, the AREG/EREG proteins that remain after silencing are still functional and sufficient to allow for the molecular function of Ctx. These findings may also explain why a four-gene classifier performs better than *AREG/EREG* status alone in predicting the likelihood of clinical benefit from Ctx in mCRC (Baker *et al*, 2011). Moreover, the secretion of AREG/EREG and the subsequent activation of members of the HER1/2/3/4 network (e.g., an AREG–EREG/EGFR autocrine loop) are necessary molecular events for AREG/EREG-promoted sensitisation to the Ctx-induced inhibition of EGFR. When AREG and/or EREG were exogenously added as recombinant proteins, Ctx sensitivity was only partially recovered in A431-derived cell lines stably expressing shRNAs specific for AREG and EREG and in pooled populations of Ctx-resistant A431 cells (data not shown). These findings confirmed earlier studies showing that the interaction between HER ligands used as exogenous recombinant proteins and HER receptors may differ from that of the membrane-anchored constitutively expressed HER ligands (Agus *et al*, 2002; Yuste *et al*, 2005). We previously reported that the combination of chemotherapy with the anti-HER2 antibody trastuzumab produced a synergistic cytotoxic interaction in HER2-negative breast cancer cells endogenously overexpressing the HER3/HER4 ligand heregulin-*β*1 (HRG*β*1) (Menendez *et al*, 2006). Conversely, trastuzumab co-treatment was ineffective at enhancing the chemotherapy efficacy in HER2-negative cancer cells exogenously treated with recombinant HRG*β*1 or in cancer cells engineered to overexpress a structural deletion mutant lacking the transmembrane domain of HRG*β*1 that, therefore, could be secreted, thus preventing the autocrine actions of HRG*β*1 via the HER receptors (Menendez *et al*, 2005, 2006).

Nevertheless, our findings confirmed not only that the minimal expression of *AREG/EREG* might identify wt *KRAS* tumours that have a high likelihood of resistance to Ctx but also suggested that the positive selection of Ctx-resistant tumour cells exhibiting AREG/EREG cross-suppression most likely has an important role in determining the emergence of Ctx resistance. Additionally, our results also show that kinase-switching phenomena can contribute to bypass the loss of EGFR-ligands signalling caused by Ctx because aberrant overactivation of FGFR3 rapidly and efficiently bypasses EGFR-dependency of SCC A431 cells upon loss of AREG/EREG. The extensive decrease in the baseline expression of AREG/EREG proteins in the A431 cells remaining after acute short-term treatment with Ctx was noteworthy. As the Ctx-induced cross-suppression of AREG/EREG was concomitant with a reduction in A431 cell numbers, we concluded that Ctx-induced cell death is not an obligatory outcome of Ctx-induced cross-suppression of AREG/EREG expression. FGFR3 activation concurrently contributes to cell survival of Ctx-naive EGFR ligands-positive SCC tumour cells challenged to Ctx at first, thus limiting its activity and likely promoting their own resistance if Ctx-induced blockade of EGFR-ligands signalling persists. This scenario agrees with the suggestion that cancer cells undergo a rapid and reversible response to diverse growth inhibitors, including RTK inhibitors, that results in drug resistance (Sharma *et al*, 2010). This Ctx-tolerant subpopulation can be selectively ablated by treatment with a selective FGFR3 inhibitor, potentially yielding a therapeutic opportunity to enhance Ctx efficacy by impairing the dynamic regulation of phenotypic heterogeneity in Ctx resistance. As immunoblotting procedures confirmed that Ctx-induced phospho-activation of FGFR3 was accompanied by increased steady-state FGFR3 protein expression (data not shown), our findings confirm and extend a recent study by Ware *et al* (2010) demonstrating that NSCLC cell lines rapidly acquire resistance to EGFR TKIs and Ctx through transcriptional de-repression of FGFR2 and FGFR3 expression. It is noteworthy that while the well-recognised mechanisms of delayed resistance to EGFR inhibitors (e.g., accumulation of second-site point mutations, gene amplification -c-Met- or increased activation of distinct RTKs -IGF-1R-) may take weeks to months (Engelman and Janne, 2008; Zhang and Chang, 2008), mechanisms of rapid resistance such as loss of EGFR-ligands and de-repression of alternative growth factor receptor pathways (e.g., FGFR3) as detailed herein can operate within few days (Ware *et al*, 2010). When A431 cells were chronically grown in medium containing high doses of Ctx (up to 200 *μ*g ml^−1^), resistance emerged within 4 to 6 weeks and cells slowly resumed their growth. However, when the small-molecule FGFR3 selective TKI PD173074 was added to Ctx during selection, prolonged exposure to the drugs resulted in the death of all cells (data not shown). These findings, altogether, suggest that Ctx-responsive, wt KRAS SCC cancer cell populations employ a dynamic survival strategy in which individual cells transiently adopt a reversible Ctx-tolerant state that efficiently protects the population from eradication by potentially lethal exposures. At later stages of continuous exposure to high-dose Ctx, when a strong dependency upon EGFR ligands signalling is definitely lost due to AREG/EREG cross-suppression, Ctx-resistant A431 cells acquire onco-addiction to the FGFR3 pathway as an additional input into MEK/ERK activation for survival and proliferation and, therefore, FGFR3 inhibition becomes necessary to circumvent refractoriness to Ctx. Of note, the EGFR molecule itself remains intact, as Ctx-resistant A431 cells overexpress EGF (Oliveras-Ferraros *et al*, 2012), they show robust tyrosine phosphorylation on several residues (Oliveras-Ferraros *et al*, 2011a), and they retain an exquisite sensitivity to the growth-inhibitory effects of the mono-EGFR TKIs gefitinib and erlotinib (Oliveras-Ferraros *et al*, 2011a).

While further studies are required to definitively address whether Ctx-induced non-viable cells express the highest levels of AREG/EREG and/or the lowest levels of FGFR3 activity, it is reasonable to suggest that as the AREG/EREG cross-suppressive mechanism allows for a rapid cancer cell *camouflage* from Ctx molecular functioning, cross-suppression of AREG/EREG may facilitate survival in Ctx-treated tumour cells, likely promoting their own resistance if the Ctx-induced EGFR blockade persists. Cellular changes associated with the loss of AREG/EREG rapidly alter the dependence of SCC carcinoma cells on EGFR signalling networks for proliferation and survival such that Ctx treatment allows for FGFR3 activation, thus suggesting that FGFR3 is repressed downstream of EGFR-AREG/EREG signalling. Accordingly, EGFR-positive A431 cell populations that are chronically adapted to grow in the presence of Ctx constitutively exhibit Ctx-unresponsive low levels of AREG/EREG and constitutive hyperactivation of FGFR3. If AREG/EREG expression is sufficient and necessary to allow for the function of Ctx against EGFR-positive wt *KRAS* tumour cells, then we may hypothesise that chronic exposure to Ctx will negatively select for initially dominant AREG/EREG-positive (Ctx-sensitive) cells and promote the selection of Ctx-resistant tumour sub-populations exhibiting a downregulated AREG/EREG-driven EGFR signalling cascade and, therefore, Ctx-unresponsive FGFR3-stimulated signalling through the ERK pathway. In this scenario, the most significant distinction between the *de novo* (primary) and acquired (secondary) resistance to Ctx might be the timing of detection. The *KRAS* mutation test informs of Ctx responsiveness solely in mCRC. However, the ability to correctly establish the AREG/EREG-FGFR3 expression/activation status before and after Ctx-based therapies might better identify wt *KRAS* tumours with a high likelihood of Ctx response not only in the treatment of mCRC but also as a monotherapy or in combination with radiotherapy for recurrent or metastatic NSCLC after failing platinum-based chemotherapy and in SCC such as locally or regionally advanced HNSCC. New AREG/EREG-based predictive tests are urgently needed to identify wt *KRAS* cancer patients that may rapidly progress on Ctx owing to the selection of Ctx-resistant AREG/EREG-negative cells. Additionally, one valuable clinical scenario would involve the simultaneous or consecutive use of PD173074-like drugs to prevent or delay overcoming of Ctx-induced growth inhibition mediated by the rapid de-repression of FGFR3 signalling that occurs in response to AREG/EREG cross-suppression.
